# Characterization of sepsis inflammatory endotypes using circulatory proteins in patients with severe infection: a prospective cohort study

**DOI:** 10.1186/s12879-022-07761-0

**Published:** 2022-10-08

**Authors:** Isis Ricaño-Ponce, Anca-Lelia Riza, Aline H. de Nooijer, Andrei Pirvu, Stefania Dorobantu, Adina Dragos, Ioana Streata, Mihaela Roskanovic, Inge Grondman, Florentina Dumitrescu, Vinod Kumar, Mihai G. Netea, Mihai Ioana

**Affiliations:** 1 Department of Internal Medicine and Radboud Center for Infectious Diseases, Radboud University Medical Center, 6500 HB Nijmegen, The Netherlands; 2 Human Genomics Laboratory, University of Medicine and Pharmacy of Craiova, Craiova, Romania; 3 Regional Centre of Medical Genetics Dolj, County Clinical Emergency Hospital Craiova, Craiova, Romania; 4 Hospital for Infectious Diseases and Pneumology “Victor Babeş” Craiova, Craiova, Romania; 5 Infectious Disease Department, University of Medicine and Pharmacy of Craiova, Craiova, Romania; 6 Department of Genetics, University Medical Center Groningen, University of Groningen, Groningen, The Netherlands; 7 Immunology and Metabolism, Life and Medical Sciences Institute, University of Bonn, Bonn, Germany

**Keywords:** Sepsis, Proteomics, Endotypes, Hyperinflammation, Severe infections

## Abstract

**Background:**

Sepsis is a heterogeneous syndrome due to a variable range of dysregulated processes in the host immune response. Efforts are made to stratify patients for personalized immune-based treatments and better prognostic prediction. Using gene expression data, different inflammatory profiles have been identified. However, it remains unknown whether these endotypes mirror inflammatory proteome profiling, which would be more feasible to assess in clinical practice. We aim to identify different inflammatory endotypes based on circulating proteins in a cohort of moderately ill patients with severe infection (Sepsis-2 criteria).

**Methods:**

In this prospective study, 92 inflammatory plasma markers were profiled using a targeted proteome platform and compared between patients with severe infection (Sepsis-2 criteria) and healthy controls. To identify endotypes with different inflammatory profiles, we performed hierarchical clustering of patients based on the differentially expressed proteins, followed by clinical and demographic characterization of the observed endotypes.

**Results:**

In a cohort of 167 patients with severe infection and 192 healthy individuals, we found 62 differentially expressed proteins. Inflammatory proteins such as TNFSF14, OSM, CCL23, IL-6, and HGF were upregulated, while TRANCE, DNER and SCF were downregulated in patients. Unsupervised clustering identified two different inflammatory profiles. One endotype showed significantly higher inflammatory protein abundance, and patients with this endotype were older and showed lower lymphocyte counts compared to the low inflammatory endotype.

**Conclusions:**

By identifying endotypes based on inflammatory proteins in moderately ill patients with severe infection, our study suggests that inflammatory proteome profiling can be useful for patient stratification.

**Supplementary Information:**

The online version contains supplementary material available at 10.1186/s12879-022-07761-0.

## Background

Severe infections are often accompanied by disturbances in the immune responses and sepsis, defined as a dysregulated host response to an infection, remains an important global health problem [1]. Despite the introduction of antibiotic treatment and supportive care in the Intensive Care Unit (ICU), sepsis still leads to an estimated 49 million cases and 11 million deaths globally every year [2]. Because of this high morbidity and mortality, the identification of criteria and biomarkers to identify and predict disease severity is crucial, in order to stratify patients for adjunctive therapies. A first approach was to use the systemic inflammatory response syndrome (SIRS) criteria that classified sepsis patients based on the systemic host response, named the Sepsis-2 criteria. However, the limited sensitivity and specificity of SIRS criteria for adverse clinical outcomes, and the increased understanding of the sepsis pathophysiology, led to the implementation of the sequential organ failure assessment (SOFA) criteria, focusing on the organ dysfunction, named the Sepsis-3 criteria [1]. However, due to the highly heterogeneous host response to sepsis, early prediction of prognosis remains challenging.

In addition to clinical classification, molecular stratification based on gene expression profiling led to the identification of different sepsis patient endotypes related to mortality in the ICU [3–5]. These endotypes encompass hyperinflammatory or immunosuppressed characteristics. However, these transcriptional scoring systems are complex, have technical demands that are often not available, and still need to be validated in independent cohorts in various countries and settings. An easier approach would be the use of circulatory proteins, as they are the key regulators that execute the cellular process, and methodologies for protein biomarker analysis are much easier available [6, 7]. The identification of potential immune protein-based endotypes could contribute to patient stratification for personalized host-directed therapies.

The aim of this study is to identify different inflammatory endotypes in patients with severe infection or sepsis, based on circulating protein abundance and to describe the patient characteristics of these groups. Many studies have investigated the heterogeneity and endotypes in severely ill sepsis patients (according to the Sepsis-3 criteria) admitted to the ICU, but information is sparse about patients with severe infection or sepsis that are moderately ill, in which identification of biomarkers to predict severity would be very important. Therefore, we focused on moderately ill patients with severe infection where sepsis was diagnosed according to the Sepsis-2 criteria that were admitted to the clinical wards of a Romanian university hospital.

## Methods

### Study design and patients

All participants had an Eastern European ancestry and were enrolled between May 2017 and November 2019. Patients were admitted to the Hospital for Infectious Diseases and Pneumology “Victor Babeş” Craiova, Romania, an academic hospital serving Dolj county in south-west Romania. Inclusion criteria were: subjects 18 years of age and older with a diagnosis of sepsis according to the ACCP/SCCM Consensus Conference criteria (Sepsis-2 criteria) [8]. Not enough information on SOFA scores was available for the establishment of Sepsis-3 criteria. Exclusion criteria were diagnosis of inherited immunodeficiency or conditions potentially leading to acquired immunodeficiency (HIV, chemotherapy or prolonged steroid treatment). As controls, healthy adult individuals were recruited at the Human Genomics Laboratory, University of Medicine and Pharmacy of Craiova. We considered healthy an individual with negative medical history, under no prescribed or self-administered medication. According to the Sepsis-2 criteria, severe sepsis was diagnosed if the patient had signs of organ dysfunction such as respiratory failure (diffuse bilateral consolidations and PaO2/FiO2 < 200 mmHg), kidney failure (urine output < 0.5 ml/kg/h), organ ischemia (pH < 7.30 or base deficit > 5 mEq/l or lactate twice the normal limit), abnormal hemostasis (platelets < 100 × 109/L or INR > 1.5) or shock (systolic blood pressure < 90 mmHg or MAP < 60 mmHg or the need for fluid resuscitation or vasopressors). Since a cohort of patients with sepsis diagnosed according to the Sepsis-2 is very different from patients with sepsis diagnosed according to the Sepsis-3 criteria, we identified this cohort as patients with severe infection in order to avoid possible confusion.

### Plasma collection and processing

Samples were collected within 24 h from Sepsis-2 diagnosis, between 07:00 and 10:00 AM and treated similarly. EDTA plasma was separated within 4 h since collection and stored at − 80 °C. Sample processing and bio archiving was performed at the Human Genomics Laboratory, University of Medicine and Pharmacy of Craiova. The sample collection was performed before initiation of antibiotic therapy.

### Targeted proteomics assays

Circulatory inflammatory proteins were measured in plasma using the targeted Olink INFLAMMATION panel (v.3021, 92 proteins) with a proximity extension assay (PEA) used by OLINK proteomics [9]. Protein concentrations were reported as log2 transformation in a normalized protein expression (NPX) scale. For further analysis, proteins detected in less than 75% of the samples were removed, as well as samples that deviate more than 0.3 NPX from the median. Protein with concentrations below the limit of detection were replaced by the lower limit of detection per protein.

### Immunoassays

In addition to the Olink proteomics platform, we decided to obtain more precise information on the concentration of a number of crucial parameters of the IL-1/IL-6 pathway involved in the pathophysiology of the hyperinflammation that characterizes some patients with severe infection or sepsis [10]. Circulating concentrations of ferritin, interleukin (IL)-6, IL-1 receptor antagonist (IL-1RA), IL-18, and IL-18 binding protein (IL-18BP) were measured with Ella Simple Plex Cartridge Kits (ProteinSimple, San Jose, USA) according to the manufacturer’s protocol. Lower limits of detection were 166 pg/mL, 2.8 pg/mL, 73.7 pg/mL, 9.6 pg/mL, and 28.4 pg/mL, for ferritin, IL-6, IL-1RA, IL-18, and IL-18BP respectively.

### Statistical analysis

The protein abundance between patients with severe infection and healthy individuals was performed in the R package limma [11] by applying a linear model correcting for age, sex and body mass index (BMI). The results from the differential abundance analysis were corrected for multiple testing using the Benjamini–Hochberg method, and FDR < 0.05 was considered statistically significant. Protein concentrations were correlated with blood cell counts using Spearman’s rank-order correlation. Cell counts were normalized using inverse rank transformation. Moderate correlation was defined as a r > 0.3 or r < − 0.3.

To identify patients endotypes based on similar protein profiles, unsupervised hierarchical clustering was performed using the Broad Institute Morpheus software (https://software.broadinstitute.org/morpheus/). The 62 proteins that were found differentially regulated between patients with severe infection and healthy individuals were used as an input. The parameters used for the hierarchical clustering were as follow: “Euclidean distance” as metric, “Average” as linkage method and clustered by rows and columns. For the comparison between the two endotypes, continuous parametrical data was analyzed using Welch two sample t-test and continuous non-parametrical data with Mann–Whitney U test. Categorical data was analyzed either by Fisher exact test or Pearson's Chi-squared test. The concentration of IL-6, IL-1RA, IL-18, IL-18BP and ferritin were normalized by inverse rank transformation. All statistical analysis were performed in R version 4.0.2.

## Results

The FUSE cohort included in this analysis comprises 192 healthy individuals and 167 patients with severe infection (Sepsis-2 criteria). Patient characteristics of both groups are shown in Table 1. In general, patients with severe infection were significantly older, had lower BMI, and had more comorbidities than healthy individuals. The most common types of infection in the patients were community-acquired pneumonia and pyelonephritis.

**Table 1:** Patient characteristics

	Healthy individuals (n = 192)	Patients with severe infection (n = 167)	*p*-value
Age (years)	44 (33–52)	63 (38–75)	**< 0.001**
Gender (n, %)			
Male	81 (42)	76 (46)	0.594
Female	111 (58)	91 (54)	
BMI (kg/m^2^)	26.2 (23.3–28.7)	24.6 (21.5–27.1)	**< 0.001**
Comorbidities			
Hypertension	20 (10)	84 (50)	**< 0.001**
Cardiovascular disease	16 (8)	91 (54)	**< 0.001**
Diabetes mellitus	3 (2)	30 (18)	**< 0.001**
Renal disease	5 (3)	23 (14)	**0.001**
Malignancy	1 (0.5)	16 (10)	**< 0.001**
Type of infection (n, %)			
CAP	NA	46 (28)	NA
VAP		1 (0.6)	
Pyelonephritis		44 (26)	
Abdominal infection		6 (4)	
Primary bacteremia		2 (1)	
Other		93 (56)	
Clinical and laboratory parameters			
Temperature (°C)	NA	38.4 (38.2–38.5)	
Mean arterial pressure (mmHg)		110 (92–120)	NA
Heart rate (bpm)		100 (92–110)	
Respiratory rate (bpm)		19 (17–22)	
Creatinine (µ/L)		83 (65–116)	
Leukocyte count (× 10^9^)		15.0 (12.8–19.3)	
Lymphocytes (× 10^9^)		1.5 (0.9–2.2)	
28-day mortality (n, %)	0 (0)	3 (1.8)	0.099

Bold values indicate significant* p*-values < 0.05

Data are presented as median (IQR) or n (%).*BMI* body mass index, *CAP* community acquired pneumonia, *VAP* ventilator-associated pneumonia, *NA* not applicable

### Inflammatory response in sepsis

**Fig. 1:**
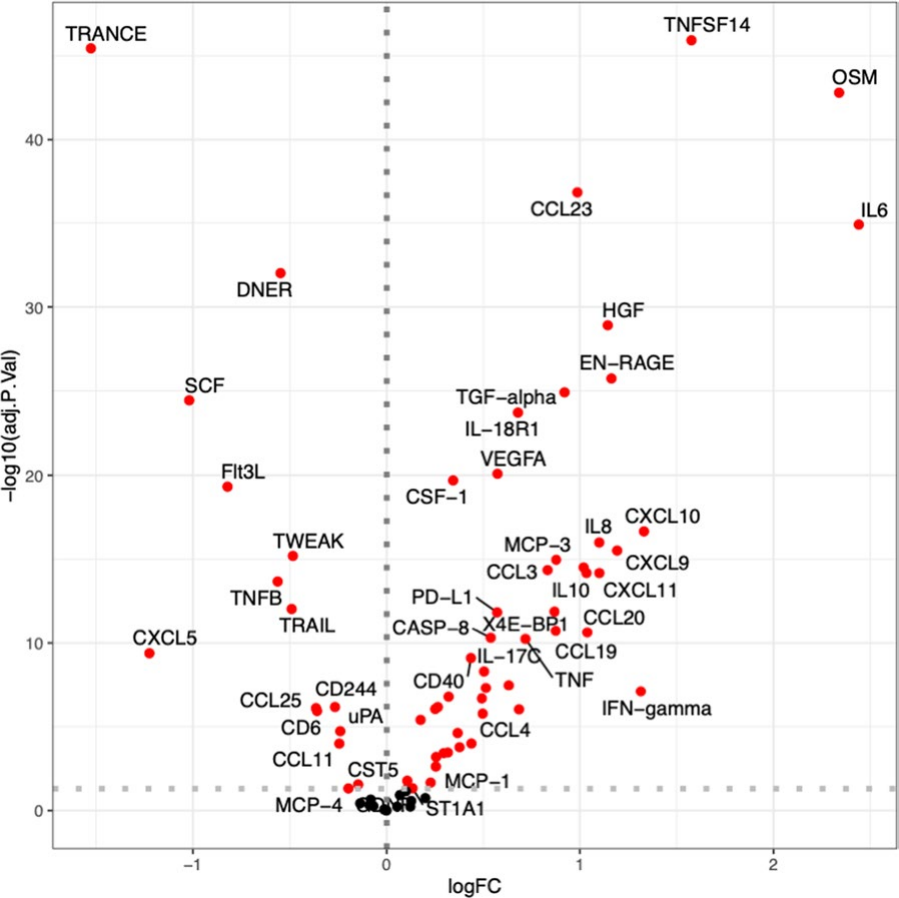
Comparison of circulatory inflammation proteins in patients with severe infection. Volcano plot with the comparison of 75 circulating proteins between patients and controls. Significant changes are depicted in red. Benjamini–Hochberg method was used to correct for multiple testing. Significance was defined as adjusted p values < 0.05. Age, sex and BMI were used as covariates in the analysis. *logFC* logarithm of the fold change

In order to examine the inflammatory profile of patients with severe infections compared to healthy individuals, 92 circulatory inflammatory markers were measured in plasma. Out of the 92 proteins tested, 75 proteins were detected in at least 75% of the samples and were included for further analysis. The protein abundance between cases (patients with severe infection) and controls (healthy individuals) was compared using a linear model correcting for age, sex and BMI. The majority of proteins (62 out of 75) were differentially expressed in patients compared to healthy individuals (Fig. 1 and Additional file 2: Table S1), confirming the known modulation in inflammatory markers during infections.

Patients with severe infection present higher concentrations of inflammatory proteins compared to controls, with 47 significant proteins reaching statistical significance. The strongest upregulated proteins were tumor necrosis factor ligand superfamily member 14 (TNFSF14) (adjusted p value = 1.21 × 10^–46^, logFC = 1.57), Oncostatin-M (OSM) (adjusted p value = 1.61 × 10^–43^, logFC = 2.33), C–C motif chemokine 23 (CCL23) (adjusted p value = 1.44 × 10^–37^, logFC = 0.98), Interleukin-6 (IL-6) (adjusted p value = 1.23 × 10^–35^, logFC = 2.44) and hepatocyte growth factor (HGF) (adjusted p value = 1.23 × 10^–29^, logFC = 1.14). Interestingly however, there were also fifteen proteins that were downregulated in patients with severe infection compared to healthy controls. The strongest were TNF-related activation-induced cytokine (TRANCE, also known as TNFSF11, RANKL, OPGL) (adjusted p value = 3.65 × 10^–46^, logFC = − 1.52), Delta and Notch-like epidermal growth factor-related receptor (DNER) (adjusted p value = 9.7 × 10^–33^, logFC = − 0.54), and the stem cell factor (SCF) (adjusted p value = 3.65 × 10–25, logFC = − 1.02). We observe a strong positive correlation of pro-inflammatory cytokines (IL-6, IL-8, IL-18, TNF) and anti-inflammatory cytokines (IL-7 and IL-10), both in patients and controls (Additional file 1: Figure S1), with all of them being upregulated in the patients. On the other hand, negative correlations between inflammatory mediators and SCF, TWEAK, TRANCE are observed: as these mediators are important for lymphopoiesis, this suggests that high inflammation is associated with inhibition of lymphopoiesis, and observation earlier made in severe COVID-19 as well [12].

### Correlation of inflammatory proteins with blood cell counts

We next correlated the concentrations of inflammatory proteins with the different types of immune cell populations in patients with severe infection and healthy individuals. While several proteins showed moderate correlation in patients at the time of admission, only a few presented weak correlations in healthy individuals. The concentration of three proteins showed moderate positive correlation with total leucocyte numbers in patients: TNFSF14 (r = 0.38), OSM (r = 0.44) and HGF (r = 0.38). Concentrations of five proteins showed moderate negative correlation with lymphocyte numbers: TNFSF14 (r = − 0.37), OSM (r = -0.40), CCL23 (r = − 0.34), and Transforming growth factor-alpha (TGF-α) (r = − 0.38), and one showed moderate positive correlation with lymphocytes: TRANCE (r = 0.36). The concentration of two proteins were moderately correlated with neutrophils OSM (r = 0.31) and TRANCE (r = − 0.39). Most of the correlated proteins are the top hits in the differential abundance analysis, all except TRANCE had higher circulating concentrations in patients compared to controls.

### Inflammatory endotypes in patients with severe infection

**Fig. 2:**
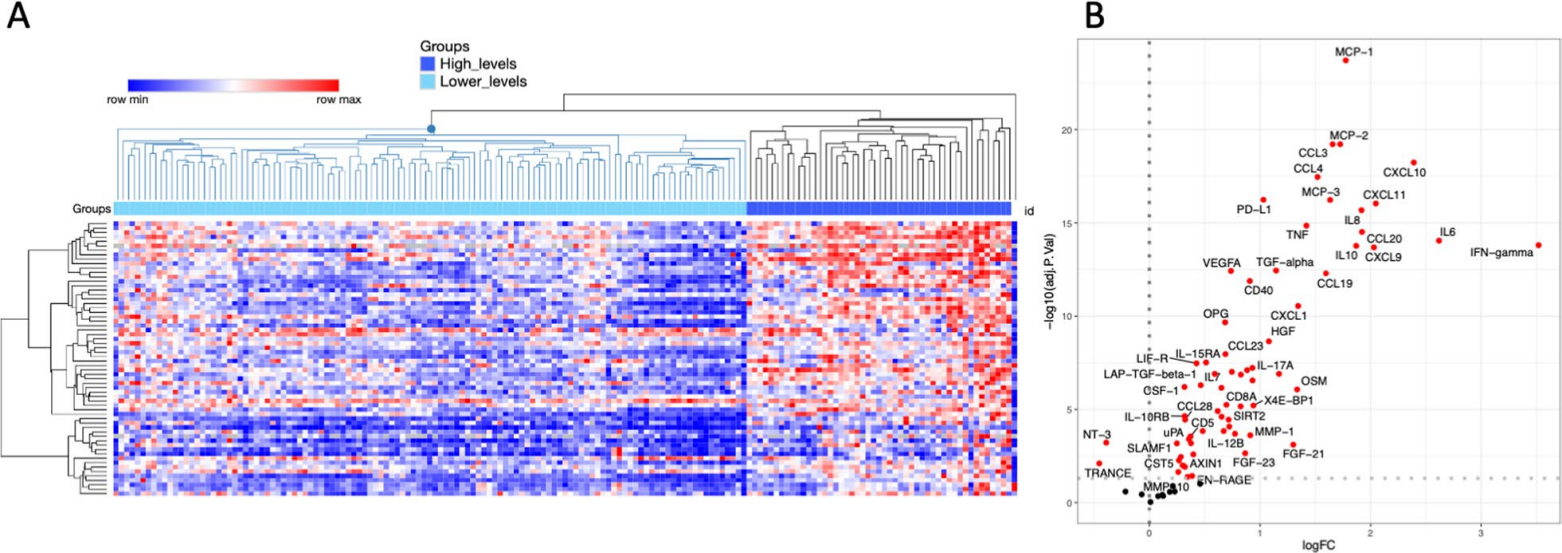
Characterization of inflammatory endotypes. **A** Hierarchical clustering of patients based on 62 proteins. **B** volcano plot of 75 circulating proteins comparing both endotypes. The 65 significantly differentially expressed proteins are highlighted in red (adjusted p values < 0.5). Age, sex and BMI were included as covariates in the analysis

Next, we assessed whether patients with severe infection could be stratified in different inflammatory endotypes, using the 62 differentially abundant proteins between patients and healthy controls. To achieve this, we performed unsupervised hierarchical clustering including only the patients (Fig. 2A). We identified two main patient clusters (n = 49 and n = 117 individuals). Only one patient did not cluster within these two main endotypes. To identify the differences between the groups, a protein differential abundant analysis was performed on the 75 high-quality proteins including age, sex and BMI as covariates (Fig. 2B and Additional file 2: Table S2). The first patient inflammatory endotype showed significantly higher protein abundance (63 upregulated proteins) compared to the second patient endotype, indicating endotype 1 as ‘high inflammatory’ and endotype 2 as ‘low inflammatory’. Only two proteins showed significantly higher concentrations in the second endotype: neurotrophin-3 (NT-3) and TRANCE.

To further characterize the two different inflammatory endotypes, we assessed the differences in demographic and clinical parameters (Table 2). The patients in the high inflammatory endotype were significantly older than in the low inflammatory endotype (median 73 vs 53 years respectively, p-value < 0.001). Additionally, hypertension (p value = 0.002) and cardiovascular disease (p value = 0.006) were more prevalent in the high inflammatory endotype. There were no significant differences in sex, BMI or the prevalence of other comorbidities. Overall, both endotypes presented similar types of infections, except for abdominal infection that was significantly more prevalent in the high inflammatory endotype (10% vs 1%, p value = 0.009). While there were no significant differences in clinical parameters, due to the relatively mild clinical picture in the majority of the patients, the high inflammatory endotype showed more abnormal laboratory parameters mirroring organ dysfunction. The incidence of abnormal renal function (based on a creatinine clearance < 60 mL/min/1.73 m^2^), abnormal liver function (based on AST or ALT twice the upper limit of the ‘normal’ value), and hematological disturbances reflected by lymphopenia (defined as lymphocytes < 1 × 10^9^/L) were higher in the high inflammatory endotype (p value =  < 0.001, 0.020, < 0.001, respectively), whereas there was no difference in leukocytosis (defined as leukocytes > 10 × 10^9^/L; p value = 0.273).

**Table 2:** Characteristics of patients with severe infections clustered for protein expression

	Endotype 1 High inflammation (n = 49)	Endotype 2 Low inflammation (n = 117)	*p*-value
Age (years)	73 (65–78)	53 (34–70)	** < 0.001**
Gender (n, %)			
Male	24 (49)	52 (44)	0.612
Female	25 (51)	65 (56)	
BMI (kg/m^2^)	24.6 (20.9–26.8)	24.6 (21.7–27.5)	0.527
Comorbidities			
Hypertension	34 (69)	49 (42)	**0.002**
Cardiovascular disease	35 (71)	55 (47)	**0.006**
Diabetes mellitus	11 (24)	18 (16)	0.257
Renal disease	5 (11)	18 (16)	0.618
Malignancy	8 (17)	8 (7)	0.075
Type of infection (n, %)			
CAP	12 (25)	34 (29)	0.704
VAP	0 (0)	1 (1)	1.000
Pyelonephritis	12 (25)	32 (27)	0.847
Abdominal infection^1^	26 (53)	41 (35)	**0.038**
Primary bacteremia	1 (2)	1 (1)	0.504
Other	8 (16)	32 (27)	0.165
Clinical parameters			
Temperature (°C)	38.4 (38.2–38.6)	38.4 (38.2–38.5)	0.594
Mean arterial pressure (mmHg)	109 (87–120)	110 (98–120)	0.382
Heart rate (bpm)	100 (94–115)	98 (91–107)	0.052
Respiratory rate (bpm)	20 (17–22)	19 (16–22)	0.595
Laboratory parameters			
Abnormal renal function (n, %)^2^	31 (66)	26 (23)	**< 0.001**
Creatinine (µmol/L)	110 (82–151)	77 (65–98)	**< 0.001**
Creatinine clearance (mL/min/1.73 m^2^)	49.3 (36.6–78.8)	78.7 (62.1–99.1)	**< 0.001**
Abnormal liver function (n, %)^3^	9 (18)	7 (6)	**0.020**
AST (U/L)	28 (18–45)	22 (14–31)	**0.009**
ALT (U/L)	26 (16–39)	22 (14–31)	0.114
Leukocytosis (n, %)^4^	42 (86)	107 (92)	0.273
Leukocyte count (× 10^9/L^)	15.0 (12.5–20.6)	15.1 (13.1–19.0)	0.731
Lymphopenia (n, %)^5^	24 (49)	24 (21)	**0.001**
Lymphocytes (× 10^9^/L)	1.1 (0.8–1.6)	1.7 (1.2–2.4)	**< 0.001**
Inflammatory parameters			
Ferritin (µg/L)	324 (180–396)	199 (126–297)	**0.002**
IL-6 (pg/mL)	91 (28–220)	11 (5–29)	**< 0.001**
IL-1RA (pg/mL)	4761 (2473–10,297)	880 (562–1292)	**< 0.001**
IL-18 (pg/mL)	414 (245–571)	229 (158–309)	**< 0.001**
IL-18BP (pg/mL)	14,421 (12,443–18,368)	8500 (6089–10,379)	**< 0.001**
Severity (n, %)^6^			
Moderate illness	34 (69)	82 (70)	1.000
Severe illness	15 (31)	35 (30)	
Organ dysfunction (n, %)			
Respiratory failure^7^	4 (8)	11 (10)	1.000
Kidney failure^8^	10 (20)	27 (24)	0.839
Organ ischemia^9^	1 (2)	7 (6)	0.439
Abnormal hemostasis^10^	2 (4)	2 (2)	0.584
Shock^11^	9 (18)	14 (12)	0.326
Length of hospital stay (days)	9 (7–13)	9 (7–12)	0.810
Resolution of severe infection (n, %)	40 (82)	108 (92)	0.056
28-day mortality (n, %)	3 (6)	0 (0)	**0.025**

Bold values indicate significant* p*-values < 0.05

Data are presented as median (IQR) or n (%).*BMI* body mass index, *CAP* community acquired pneumonia, *VAP* ventilator-associated pneumonia, *AST* aspartate aminotransferase, *ALT* alanine aminotransferase, *NA* not applicable

^1^Including *Clostridium difficile* enterocolitis. ^2^defined as creatinine clearance < 60 mL/min/1.73 m^2^. ^3^defined as AST or ALT twice the upper limit of the ‘normal’ value. ^4^defined as leukocytes > 10 × 10^9^/L. ^5^defined as lymphocytes < 1 × 10^9^/L. ^6^Moderate illness is defined as sepsis according to the Sepsis-2 criteria, severe illness is defined as severe sepsis or septic shock according to the Sepsis-2 criteria. ^7^defined as diffuse bilateral consolidations and PaO_2_/FiO_2_ < 200 mmHg. ^8^defined as urine output < 0.5 ml/kg/h. ^9^defined as pH < 7.30 or base deficit > 5 mEq/l or lactate twice the normal limit. ^10^defined as platelets < 100 × 10^9^/L or INR > 1.5. ^11^defined as systolic blood pressure < 90 mmHg or MAP < 60 mmHg or the need for fluid resuscitation or vasopressors

In line with their level of inflammation, the patients in the high inflammatory endotype showed increased circulating concentrations of ferritin (median 324 vs 199 µg/L, p value = 0.002), as well as significantly higher levels of the pro-inflammatory cytokines IL-6, IL-1RA, IL-18 and IL-18BP (p value < 0.001). The classification of severe illness and the underlying organ dysfunction were not significantly different between the two endotypes. Moreover, no differences were observed for length of hospital stay and resolution of severe infection. Three patients in this cohort died from severe infection. They were all classified in the ‘high inflammatory’ endotype, and the direct cause of death was cardiac insufficiency and respiratory failure in a 75-year-old man with severe *Staphylococcus* *aureus* and *Serratia marcescens* infection*,* cardiac and respiratory failure in a 91-year-old woman with severe *Staphylococcus aureus* infection, and renal insufficiency in an 86-year-old man with *Clostridium difficile* enterocolitis.

### Severe infections and COVID-19

**Fig. 3:**
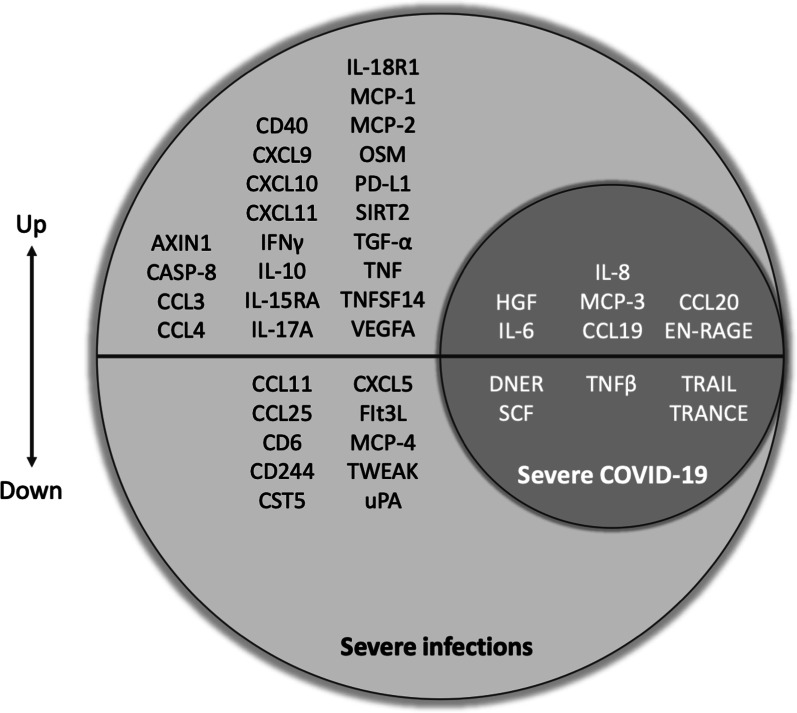
Differentially expressed inflammatory proteins in severe infections vs COVID-19 severity. In the light grey circle are the differentially abundant proteins in sepsis, in the dark grey circle the differentially abundant proteins shared by severe infections in general and COVID-19 severity specifically

The inflammatory profile identified in the patients with severe infection compared to healthy individuals resembles the inflammatory profile we previously identified specifically for COVID-19 patients with different disease severity [12]. To assess the similarities between the two diseases for the measured proteins, we extracted a list of 12 proteins that were significantly different in the critical COVID-19 patients admitted to the ICU vs the moderately ill COVID-19 patients on non-ICU wards. Interestingly, all these proteins are also differentially expressed in patients with severe infection compared to controls (Fig. 3).

## Discussion

In this cohort of moderately ill patients with severe infection admitted to the clinical wards, we identified two inflammatory endotypes based on 62 immune-related circulating proteins that are regulated differently between patients and healthy individuals. We showed that the ‘high inflammatory’ endotype consisted of older patients with more cardio-vascular comorbidities and abnormal laboratory parameters. These findings implicate that, based on circulating inflammatory proteins, stratification of moderately ill patients with severe infection is possible. The identification of a patients’ inflammatory endotype could potentially lead to improved personalized therapeutic strategies in the heterogeneous disease course of sepsis, e.g., immunotherapy.

In the past decades, the heterogeneity in sepsis has become well recognized and much effort has been paid in the identification of endotypes based on different pathophysiological mechanisms with multiple advanced *omics* technologies [5, 13]. Transcriptomic studies revealed subgroups of sepsis patients that could allow for therapeutic decisions based on the underlying biological process, if clinically feasible and rapid assessment is possible [3, 4]. However, the direct functional consequences of these transcriptomic-based approaches are not well-characterized, and the implementation in clinical practice is hampered by the unavailability of some of the complex methodologies on large scale in the hospitals. Proteomic analyses are considered as a step closer to assessing the functionality of the inflammatory effects, and protein biomarkers are often associated with the outcome [6, 14]. Here we demonstrate that stratification of moderately ill patients with severe infection based on proteome profile is possible, similar to the results of the transcriptomics studies.

Whereas most of the studies investigated endotypes in severely ill sepsis patients (according to the Sepsis-3 criteria) admitted to the ICU, we focused on a cohort of moderately ill patients with severe infection where sepsis was diagnosed according to the Sepsis-2 criteria admitted to the clinical wards. It is thus important to observe that different endotypes are also present in the milder disease and that endotype-specific treatments could be also beneficial in this population. This could potentially prevent moderately ill sepsis patients to deteriorate, although our study did not investigate this hypothesis, and future studies are warranted. In our cohort, the individuals with an endotype characterized by high inflammation were older with more cardiovascular complications, and subsequently displayed more abnormal laboratory parameters compared to the patients with less inflammation. Differences in regulation of proteins in elderly patients compared to younger sepsis controls are supported by a previous study [7], and the more severely dysregulated immune response may at least explain the poorer outcome.

This study also has limitations. First of all, the endotypes identified were studied in only one cohort of patients, and they need to be validated in an independent cohort. Second, we should also note that in this cohort patients were classified according to the Sepsis-2 criteria, due to absence of all parameters needed to calculate SOFA scores, making that this cohort differs from other studies in sepsis patients according to the Sepsis-3 definition. The most important difference between the Sepsis-2 and the Sepsis-3 definition is severity with organ disfunction and a higher disease severity in patients fulfilling the Sepsis-3 criteria. Since we explicitly aimed to study moderately ill patients with severe infections the Sepsis-2 criteria were used. According to these criteria, 50 patients were diagnosed with severe sepsis or septic shock, corresponding with a sepsis diagnosis according to the Sepsis-3 definition. However, the use of the Sepsis-2 criteria resulted in a limited number of cases with adverse clinical outcomes, such as ICU admission or mortality. Future research should focus on the different protein expression in moderately ill patients that deteriorate compared to patients that recover. Third, an important limitation of this pragmatic study performed in the general wards rather than on intensive care units, was the lack of complete clinical information to calculate severity scores, such as the acute physiology and chronic health evaluation II (APACHE II) score and the SOFA score. Due to the low number of patients that died within the study, the power to assess the effects of the endotypes on mortality was very limited. Finally, the direct application of omics-based endotype identification in challenging. However, the value of our study is much clearer at the level of pathway and biomarker identification, which teaches us important aspects on pathophysiology of severe infections and contributes to the future development of immunotherapy.

Targeted proteomics is a cost-effective technique to measure multiple proteins at the same time. In the case of the Olink platform used in the present study, the proteins present in the inflammation panel have been selected based on their function in inflammatory and innate immune processes that we know play a very important role in the mechanisms leading to immune dysregulation in sepsis. In the study, we showed increased circulating concentrations of 47 inflammatory proteins in patients with severe infection compared to healthy controls, with the most robust increase in the circulating concentration observed for TNFSF14, OSM, CCL23, IL-6, and HGF. All these proteins are involved in pro-inflammatory pathways, such as cytokine production and proliferation, recruitment, and stimulation of lymphocytes and monocytes, which confirmed the inflammatory state of sepsis and highlight the role of these pathways in the sepsis-related inflammatory response [15–18]. Next, patients with severe infection had a lower concentration of fifteen proteins among which TRANCE, DNER, and SCF were most significantly different. These proteins are involved in pathways related to hematopoietic stem cell maintenance (especially lymphopoiesis) and reduction of apoptosis [19, 20]. Negative correlations between inflammatory mediators and SCF, TWEAK, TRANCE have been observed, strongly suggesting that an ineffective systemic inflammation is associated with negative effects on the bone marrow lymphopoiesis, as earlier observed in severe COVID-19 as well [12]. Therefore, one could speculate that the lower concentrations of these proteins contribute to immune cell dysregulation, and especially lymphopenia which is a known contributor to immune paralysis in sepsis [21]. Our data further underline the association between these differentially expressed proteins and circulating immune cells. Next, it is interesting to note that we observed similarities of these differentially expressed proteins in patients with severe infection in general compared to COVID-19 severity of disease, which suggests the involvement of these pathways more broadly in severe infections. A similar analysis was performed comparing the two endotypes, indicating that 65 proteins were differentially regulated, of which 63 were upregulated and two proteins were downregulated.

Identification of inflammatory endotypes in moderately ill sepsis patients has important implications for future clinical trials with immunotherapy. If these endotypes and the hypothesis that the high inflammatory endotype has more adverse outcomes (ICU admission, mortality) are validated in independent cohorts, these patients might potentially benefit from anti-inflammatory therapies. This concept will be addressed in a clinical trial with personalized immunotherapy, targeting both hyperinflammation and immunosuppression at the clinical wards and ICUs in different European hospitals (NCT04990232).

## Supplementary Information

**Additional file 1. Supplementary figure 1**. Correlation plot of the 75 proteins included in the study in healthy controls and patients with severe infections. The correlation matrix was generated using the “psych” package in R and it was plotted using corrplot from the “rstatix” package. The colours represent the degree of pairwise correlation based on Spearman’s rank correlation coefficient. Positive correlations are depicted in red, while negative correlations are in blue. The size of the circles represents the magnitude of the correlation coefficient.

**Additional file 2.** **Supplementary tables**. **Supplementary Table 1** shows the results from the differential abundance analysis between patients with severe infections and healthy controls. **Supplementary Table 2** shows the results from the differential abundance analysis between the two endotypes.

## Data Availability

The datasets generated during and/or analyzed during the current study are available from the corresponding author on reasonable request.
