# Supplementary material for: Characterization of sepsis inflammatory endotypes using circulatory proteins in patients with severe infection: a prospective cohort study
Source: BMC Infect Dis. 2022 Oct 8;22:778. doi: 10.1186/s12879-022-07761-0 (PMC9547371; doi:10.1186/s12879-022-07761-0)
Supplement: Supplementary file 1 — Additional file 1. Supplementary figure 1. Correlation plot of the 75 proteins included in the study in healthy controls and patients with severe infections. The correlation matrix was generated using the “psych” package in R and it was plotted using corrplot from the “rstatix” package. The colours represent the degree of pairwise correlation based on Spearman’s rank correlation coefficient. Positive correlations are depicted in red, while negative correlations are in blue. The size of the circles represents the magnitude of the correlation coefficient. [file 12879_2022_7761_MOESM1_ESM.pdf]

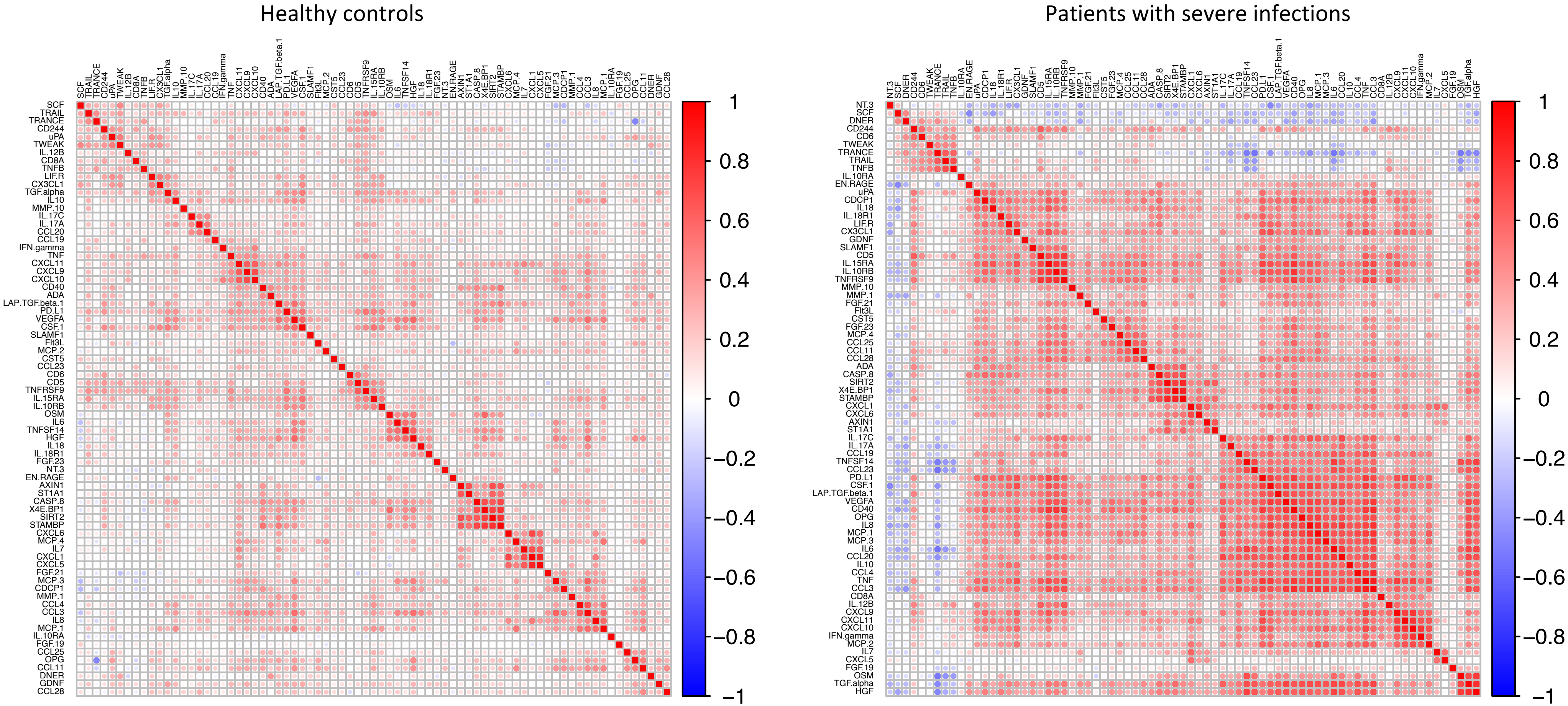

Supplementary figure 1. Correlation plot of the 75 proteins included in the study in healthy controls and patients with severe infections. The correlation matrix was generated using the “psych” package in R and it was plotted using corrplot from the “rstatix” package. The colours represent the degree of pairwise correlation based on the Spearman's rank correlation coefficient. Positive correlations are depicted in red, while negative correlations are in blue. The size of the circles represents the magnitude of the correlation coefficient.
