# Supplementary material for: Characterization of sepsis inflammatory endotypes using circulatory proteins in patients with severe infection: a prospective cohort study
Source: BMC Infect Dis. 2022 Oct 8;22:778. doi: 10.1186/s12879-022-07761-0 (PMC9547371; doi:10.1186/s12879-022-07761-0)
Supplement: Supplementary file 2 — Additional file 2. Supplementary tables. Supplementary Table 1 shows the results from the differential abundance analysis between patients with severe infections and healthy controls. Supplementary Table 2 shows the results from the differential abundance analysis between the two endotypes. [file 12879_2022_7761_MOESM2_ESM.docx]

**Supplementary Tables**

**Supplementary Table 1. Results from the differential abundance analysis between patients with severe infections and healthy controls**

| **Protein** | **logFC** | **AveExpr** | **t** | **P.Value** | **adj.P.Val** | **B** |
| --- | --- | --- | --- | --- | --- | --- |
| TNFSF14 | 1,57635662 | 4,59536159 | 17,1439123 | 1,61E-48 | 1,21E-46 | 99,5165645 |
| TRANCE | -1,5265545 | 4,37939287 | -16,953499 | 9,73E-48 | 3,65E-46 | 97,7253798 |
| OSM | 2,33856721 | 5,0861788 | 16,2632012 | 6,45E-45 | 1,61E-43 | 91,2516845 |
| CCL23 | 0,98720407 | 10,8524809 | 14,7605006 | 7,67E-39 | 1,44E-37 | 77,3178297 |
| IL6 | 2,44024978 | 4,61202529 | 14,2509919 | 8,22E-37 | 1,23E-35 | 72,6626215 |
| DNER | -0,5479017 | 9,04733752 | -13,495418 | 7,76E-34 | 9,70E-33 | 65,8448693 |
| HGF | 1,14409221 | 9,4529168 | 12,6749307 | 1,15E-30 | 1,23E-29 | 58,5811569 |
| EN-RAGE | 1,16294191 | 3,10457669 | 11,8193565 | 1,94E-27 | 1,82E-26 | 51,1951368 |
| TGF-alpha | 0,92109032 | 3,89760451 | 11,582219 | 1,46E-26 | 1,22E-25 | 49,1870525 |
| SCF | -1,0191748 | 8,46337345 | -11,440425 | 4,86E-26 | 3,65E-25 | 47,9951496 |
| IL-18R1 | 0,67924975 | 8,83720398 | 11,2288577 | 2,88E-25 | 1,96E-24 | 46,2294847 |
| VEGFA | 0,57374971 | 10,7624073 | 10,2008596 | 1,29E-21 | 8,05E-21 | 37,8873348 |
| CSF-1 | 0,34328678 | 10,2422951 | 10,2798361 | 3,47E-21 | 2,00E-20 | 37,0500191 |
| Flt3L | -0,821662 | 9,05644641 | -9,9582362 | 8,81E-21 | 4,72E-20 | 35,9811491 |
| CXCL10 | 1,33135622 | 9,95361298 | 9,15203322 | 4,35E-18 | 2,17E-17 | 29,8402559 |
| IL8 | 1,10011431 | 5,2754383 | 8,93927555 | 2,12E-17 | 9,95E-17 | 28,2725597 |
| CXCL9 | 1,19228427 | 7,82896557 | 8,78047723 | 6,82E-17 | 3,01E-16 | 27,1177278 |
| TWEAK | -0,4841269 | 9,62861253 | -8,6721143 | 1,50E-16 | 6,26E-16 | 26,3373452 |
| MCP-3 | 0,87707229 | 2,72811058 | 8,5917193 | 2,69E-16 | 1,06E-15 | 25,7624621 |
| IL-17A | 1,01946291 | 2,74041332 | 8,43826632 | 8,15E-16 | 3,06E-15 | 24,6674468 |
| CCL3 | 0,83196445 | 5,47581357 | 8,37912542 | 1,23E-15 | 4,40E-15 | 24,2593507 |
| IL10 | 1,03431093 | 4,9042927 | 8,31492478 | 1,94E-15 | 6,62E-15 | 23,8103875 |
| CXCL11 | 1,10062825 | 8,11254554 | 8,30824489 | 2,03E-15 | 6,64E-15 | 23,7638081 |
| TNFB | -0,5627784 | 4,63468813 | -8,1361219 | 6,81E-15 | 2,13E-14 | 22,572371 |
| TRAIL | -0,4904998 | 7,7893022 | -7,5771837 | 3,05E-13 | 9,16E-13 | 18,8240212 |
| X4E-BP1 | 0,86792981 | 7,72374292 | 7,5163585 | 4,57E-13 | 1,32E-12 | 18,4276282 |
| PD-L1 | 0,5716774 | 6,95829621 | 7,4955397 | 5,24E-13 | 1,46E-12 | 18,2924867 |
| CCL19 | 0,87449908 | 9,77167763 | 7,09500464 | 6,98E-12 | 1,87E-11 | 15,7464628 |
| CCL20 | 1,03846807 | 7,87990688 | 7,05574258 | 8,95E-12 | 2,31E-11 | 15,5025158 |
| CASP-8 | 0,53886788 | 2,5263379 | 7,00057995 | 1,95E-11 | 4,89E-11 | 14,8660524 |
| TNF | 0,71789775 | 3,71566384 | 6,89965084 | 2,38E-11 | 5,75E-11 | 14,5428356 |
| CXCL5 | -1,2247225 | 8,21397571 | -6,5729095 | 1,75E-10 | 4,09E-10 | 12,5876067 |
| CD40 | 0,43509059 | 11,6208273 | 6,45763298 | 3,47E-10 | 7,89E-10 | 11,9154973 |
| IL-17C | 0,50443987 | 2,61057669 | 6,13407929 | 2,27E-09 | 5,01E-09 | 10,0797541 |
| SIRT2 | 0,63174356 | 3,49203766 | 5,7859665 | 1,58E-08 | 3,38E-08 | 8,19044601 |
| IL18 | 0,51369556 | 8,63196262 | 5,71339309 | 2,33E-08 | 4,86E-08 | 7,80804679 |
| IFN-gamma | 1,31493414 | 7,24666267 | 5,62089215 | 3,83E-08 | 7,76E-08 | 7,32648131 |
| OPG | 0,3199969 | 10,5477712 | 5,47333227 | 8,32E-08 | 1,64E-07 | 6,57194303 |
| CDCP1 | 0,49239134 | 3,76427685 | 5,42416472 | 1,07E-07 | 2,07E-07 | 6,32429281 |
| LAP-TGF-beta-1 | 0,26434362 | 7,30338318 | 5,18524804 | 3,62E-07 | 6,76E-07 | 5,14804251 |
| CD244 | -0,266696 | 6,94308986 | -5,1808679 | 3,70E-07 | 6,76E-07 | 5,12690149 |
| CCL25 | -0,3647736 | 6,16668802 | -5,1455373 | 4,41E-07 | 7,87E-07 | 4,95694081 |
| IL-15RA | 0,25085656 | 1,76925889 | 5,11644576 | 5,09E-07 | 8,88E-07 | 4,81774618 |
| MMP-1 | 0,6846748 | 9,62181947 | 5,10243171 | 5,45E-07 | 9,30E-07 | 4,75093618 |
| CD6 | -0,359267 | 5,45236652 | -5,0513808 | 7,01E-07 | 1,17E-06 | 4,50889885 |
| CCL4 | 0,49703922 | 6,27036825 | 4,97279863 | 1,03E-06 | 1,67E-06 | 4,14046152 |
| SLAMF1 | 0,17536456 | 2,09355259 | 4,78848461 | 2,46E-06 | 3,93E-06 | 3,29609951 |
| uPA | -0,2395686 | 10,315091 | -4,4420267 | 1,19E-05 | 1,86E-05 | 1,78532815 |
| AXIN1 | 0,36659785 | 2,03192554 | 4,38016727 | 1,56E-05 | 2,39E-05 | 1,52623515 |
| MCP-2 | 0,43751519 | 8,65121368 | 4,03444432 | 6,69E-05 | 0,00010042 | 0,1387327 |
| CCL11 | -0,2443624 | 6,93095465 | -4,027045 | 6,90E-05 | 0,00010146 | 0,11016987 |
| MMP-10 | 0,37629394 | 8,86693253 | 3,89606887 | 0,00011669 | 0,0001683 | -0,3874743 |
| CD8A | 0,31604226 | 10,9284823 | 3,70221013 | 0,00024742 | 0,00035012 | -1,0962297 |
| TNFRSF9 | 0,2945654 | 7,33756231 | 3,67754947 | 0,00027163 | 0,00037726 | -1,1839924 |
| CX3CL1 | 0,25544722 | 6,0719373 | 3,52727808 | 0,00047452 | 0,00064707 | -1,7069959 |
| STAMBP | 0,25407297 | 4,45521877 | 3,1465339 | 0,00179072 | 0,00239829 | -2,94051 |
| LIF-R | 0,10678501 | 4,06129142 | 2,50198299 | 0,01279571 | 0,01683646 | -4,7228459 |
| MCP-1 | 0,22717779 | 11,6708204 | 2,40301352 | 0,01676905 | 0,02168412 | -4,9618437 |
| CST5 | -0,1472546 | 5,34990872 | -2,2988462 | 0,02208938 | 0,02807973 | -5,2033 |
| ST1A1 | 0,13379934 | 1,89268086 | 2,08791619 | 0,03751216 | 0,04688378 | -5,6603544 |
| GDNF | 0,1077958 | 2,50180808 | 2,08113609 | 0,03813214 | 0,04688378 | -5,6743351 |
| MCP-4 | -0,1975527 | 12,7995871 | -2,0624468 | 0,03988659 | 0,04824991 | -5,7126431 |
| CD5 | 0,09947225 | 5,42430727 | 1,89202926 | 0,05929424 | 0,07058838 | -6,0463582 |
| IL-10RB | 0,06823302 | 6,11656128 | 1,63848233 | 0,1022001 | 0,11976575 | -6,4905935 |
| FGF-23 | 0,19976988 | 2,93778696 | 1,41897354 | 0,15677722 | 0,18089679 | -6,824384 |
| NT-3 | -0,0825568 | 3,40872735 | -1,2969737 | 0,19547595 | 0,22213176 | -6,9894084 |
| IL-12B | 0,12705841 | 6,06596262 | 1,17931309 | 0,23905708 | 0,26760121 | -7,134645 |
| CXCL1 | -0,1368481 | 7,35492774 | -0,9641572 | 0,33561827 | 0,37016721 | -7,3647736 |
| FGF-19 | -0,089204 | 8,5429383 | -0,7876181 | 0,43144154 | 0,46895819 | -7,5192586 |
| CXCL6 | -0,0690414 | 7,92990384 | -0,6731984 | 0,50125565 | 0,53705963 | -7,6028097 |
| IL7 | 0,05400395 | 2,65264758 | 0,60270301 | 0,54708806 | 0,57790993 | -7,6477866 |
| FGF-21 | 0,12248592 | 6,35174705 | 0,59002013 | 0,55554957 | 0,57869746 | -7,6553521 |
| CCL28 | -0,0127168 | 2,6359807 | -0,219017 | 0,82676166 | 0,84941266 | -7,8055325 |
| ADA | -0,0108991 | 5,36447209 | -0,1694031 | 0,86557534 | 0,87727231 | -7,8151789 |
| IL-10RA | -0,000812 | 1,56828198 | -0,008821 | 0,9929669 | 0,9929669 | -7,8295059 |
| logFC=logarithm of the fold change; AveExpr = average log-expression values; t = moderated t-statistic; P.Value = associated p-value; adj.P.Value = the p-value adjusted for multiple testing using Benjamini and Hochberg method; B = log-odds that the gene protein differentially expressed. | | | | | | |

**Supplementary Table 2. Results from the differential abundance analysis between the two endotypes**

| **Protein** | **logFC** | **AveExpr** | **t** | **P.Value** | **adj.P.Val** | **B** |
| --- | --- | --- | --- | --- | --- | --- |
| MCP-1 | 1,75541987 | 11,9498647 | 11,9933875 | 8,10E-24 | 6,08E-22 | 43,4108316 |
| MCP-2 | 1,73467671 | 8,98066148 | 10,9000684 | 7,33E-21 | 2,75E-19 | 36,6497047 |
| CXCL10 | 2,43920699 | 10,8288048 | 10,3027516 | 2,94E-19 | 7,34E-18 | 32,9868598 |
| CCL3 | 1,63356442 | 6,13538374 | 10,236521 | 4,41E-19 | 8,28E-18 | 32,5828135 |
| CXCL11 | 2,06814949 | 8,82786652 | 9,7187689 | 1,05E-17 | 1,57E-16 | 29,4424495 |
| CCL4 | 1,47420677 | 6,68521826 | 9,36588444 | 8,89E-17 | 1,11E-15 | 27,3239437 |
| IFN-gamma | 3,77052863 | 8,06459748 | 9,32491287 | 1,14E-16 | 1,22E-15 | 27,0793027 |
| PD-L1 | 1,02497247 | 7,34221342 | 9,2376798 | 1,92E-16 | 1,80E-15 | 26,5594234 |
| IL8 | 1,87167481 | 6,16170277 | 8,86935375 | 1,74E-15 | 1,45E-14 | 24,3802572 |
| MCP-3 | 1,55356239 | 3,35448316 | 8,811121 | 2,46E-15 | 1,84E-14 | 24,0382597 |
| TNF | 1,41080578 | 4,23436335 | 8,65088504 | 6,33E-15 | 4,32E-14 | 23,1010553 |
| CXCL9 | 2,09046233 | 8,68995245 | 8,60371512 | 8,36E-15 | 5,23E-14 | 22,8262766 |
| IL10 | 1,91099446 | 5,56941103 | 8,50606696 | 1,48E-14 | 8,56E-14 | 22,2591195 |
| IL6 | 2,62234412 | 6,15835981 | 8,42083817 | 2,44E-14 | 1,31E-13 | 21,7659917 |
| CCL19 | 1,68167529 | 10,2738048 | 8,23780128 | 7,09E-14 | 3,55E-13 | 20,7131925 |
| CCL20 | 1,81627919 | 8,55352871 | 8,11528218 | 1,44E-13 | 6,75E-13 | 20,0134611 |
| VEGFA | 0,7123336 | 11,1641607 | 7,6220768 | 2,39E-12 | 1,06E-11 | 17,2408536 |
| TGF-alpha | 1,09238156 | 4,50562026 | 7,47596315 | 5,42E-12 | 2,26E-11 | 16,434203 |
| CD40 | 0,87145589 | 11,9724389 | 7,3043896 | 1,40E-11 | 5,55E-11 | 15,4963345 |
| CXCL1 | 1,31162446 | 7,41039516 | 6,93291746 | 1,07E-10 | 4,00E-10 | 13,5027235 |
| OPG | 0,69693211 | 10,8512722 | 6,7763939 | 2,47E-10 | 8,81E-10 | 12,6788561 |
| LAP-TGF-beta-1 | 0,50870288 | 7,50723916 | 6,26997668 | 3,47E-09 | 1,18E-08 | 10,0852648 |
| HGF | 1,07291061 | 10,2091934 | 6,10467725 | 8,04E-09 | 2,62E-08 | 9,26420092 |
| CCL23 | 0,69679036 | 11,4508166 | 6,02725029 | 1,19E-08 | 3,70E-08 | 8,88419516 |
| LIF-R | 0,44327308 | 4,15106768 | 5,98053024 | 1,50E-08 | 4,49E-08 | 8,65634354 |
| IL-18R1 | 0,62015398 | 9,22733806 | 5,78734779 | 3,88E-08 | 1,12E-07 | 7,72604596 |
| IL-17C | 0,78381676 | 2,91519735 | 5,56683825 | 1,13E-07 | 3,11E-07 | 6,68828029 |
| IL-15RA | 0,49718512 | 1,99277652 | 5,56053672 | 1,16E-07 | 3,11E-07 | 6,65901367 |
| CX3CL1 | 0,73941445 | 6,3169371 | 5,5473442 | 1,24E-07 | 3,17E-07 | 6,59781412 |
| CXCL6 | 0,91600934 | 7,97111161 | 5,54170447 | 1,27E-07 | 3,17E-07 | 6,57168115 |
| TNFSF14 | 0,97908014 | 5,50498335 | 5,5009743 | 1,54E-07 | 3,73E-07 | 6,38347573 |
| IL7 | 0,64803384 | 2,67160129 | 5,31837553 | 3,63E-07 | 8,50E-07 | 5,55128857 |
| CSF-1 | 0,32331014 | 10,4685563 | 5,31078541 | 5,16E-07 | 1,14E-06 | 5,37375517 |
| IL-17A | 1,14641743 | 3,41807883 | 5,26880931 | 4,59E-07 | 1,04E-06 | 5,32748771 |
| OSM | 1,31944044 | 6,42334297 | 5,03624674 | 1,31E-06 | 2,81E-06 | 4,30397672 |
| TNFRSF9 | 0,74413121 | 7,59232497 | 4,87186481 | 2,72E-06 | 5,67E-06 | 3,59968286 |
| CD8A | 0,71911393 | 11,1178917 | 4,77467614 | 4,16E-06 | 8,43E-06 | 3,19132626 |
| SIRT2 | 0,81571417 | 3,86595729 | 4,65965946 | 6,81E-06 | 1,34E-05 | 2,71596909 |
| CASP-8 | 0,66967997 | 2,90019225 | 4,43747235 | 2,05E-05 | 3,94E-05 | 1,81801277 |
| CDCP1 | 0,75795772 | 4,27116323 | 4,3730534 | 2,25E-05 | 4,21E-05 | 1,56985704 |
| IL-10RB | 0,32329318 | 6,21247877 | 4,34814815 | 2,49E-05 | 4,55E-05 | 1,47291664 |
| STAMBP | 0,59638916 | 4,61616303 | 4,33201607 | 2,65E-05 | 4,70E-05 | 1,41035526 |
| X4E-BP1 | 0,89055979 | 8,28238497 | 4,32827324 | 2,69E-05 | 4,70E-05 | 1,39586625 |
| CCL28 | 0,31566332 | 2,75885465 | 4,07211492 | 7,43E-05 | 0,00012668 | 0,42784533 |
| IL18 | 0,68831596 | 8,99048568 | 3,93089635 | 0,00012758 | 0,00021264 | -0,0854967 |
| ADA | 0,48248871 | 5,38168129 | 3,79831793 | 0,00020925 | 0,00034117 | -0,5539234 |
| IL-12B | 0,7856742 | 6,18892748 | 3,7667909 | 0,00023496 | 0,00037493 | -0,6633603 |
| MCP-4 | 0,68553698 | 12,8459294 | 3,72948421 | 0,00026924 | 0,00042068 | -0,79188 |
| uPA | 0,36238998 | 10,2407941 | 3,59746555 | 0,0004325 | 0,00066198 | -1,2380678 |
| MMP-1 | 0,85301545 | 10,2165186 | 3,55725427 | 0,00049843 | 0,00074764 | -1,3712776 |
| NT-3 | -0,393428 | 3,33337613 | -3,5283296 | 0,00055159 | 0,00081116 | -1,4663125 |
| ST1A1 | 0,35742258 | 1,97123523 | 3,47562175 | 0,00066242 | 0,00095542 | -1,6377914 |
| CD5 | 0,34161172 | 5,51376626 | 3,33066595 | 0,00108463 | 0,00153485 | -2,0979637 |
| GDNF | 0,30481936 | 2,61570323 | 3,23969449 | 0,00146631 | 0,00203654 | -2,3780934 |
| SLAMF1 | 0,23914312 | 2,21439194 | 3,21326451 | 0,0015987 | 0,00218005 | -2,4582127 |
| FGF-21 | 1,18500352 | 6,71985084 | 3,11610989 | 0,00218679 | 0,00292874 | -2,7477799 |
| AXIN1 | 0,36223159 | 2,20804116 | 2,96461983 | 0,00351344 | 0,00462295 | -3,1836048 |
| CD244 | 0,28032873 | 6,80967529 | 2,8581213 | 0,00485214 | 0,00627432 | -3,4783901 |
| FGF-23 | 0,7549738 | 3,13916232 | 2,69120186 | 0,00790685 | 0,01005108 | -3,9208379 |
| CCL11 | 0,29824025 | 6,9245309 | 2,48527021 | 0,01401486 | 0,01751858 | -4,4331879 |
| CST5 | 0,26758856 | 5,38970684 | 2,29281962 | 0,02321276 | 0,02812699 | -4,8779056 |
| TRANCE | -0,3888442 | 3,44552277 | -2,2921619 | 0,02325165 | 0,02812699 | -4,8793684 |
| MMP-10 | 0,35732177 | 9,15655652 | 2,09198612 | 0,0380816 | 0,04533523 | -5,3062187 |
| EN-RAGE | 0,37223965 | 3,8309869 | 2,0015427 | 0,04709139 | 0,054603 | -5,4870064 |
| CCL25 | 0,25148032 | 6,11443381 | 1,99942319 | 0,0473226 | 0,054603 | -5,4911523 |
| CD6 | 0,24905803 | 5,25953265 | 1,76231815 | 0,08000071 | 0,09090989 | -5,9284638 |
| CXCL5 | 0,44012804 | 7,60677032 | 1,58344573 | 0,11537264 | 0,12914848 | -6,2232902 |
| Flt3L | 0,25863864 | 8,71036006 | 1,53460042 | 0,12693517 | 0,14000203 | -6,2985005 |
| TNFB | 0,12667464 | 4,31031206 | 0,97666743 | 0,33026647 | 0,3589853 | -6,9938769 |
| SCF | -0,170225 | 7,83965587 | -0,8893693 | 0,37519262 | 0,4019921 | -7,0751343 |
| TWEAK | 0,09188624 | 9,37613987 | 0,83133539 | 0,4070721 | 0,42424059 | -7,1249879 |
| IL-10RA | 0,14059359 | 1,61221065 | 0,83098224 | 0,40727097 | 0,42424059 | -7,125281 |
| FGF-19 | 0,13658581 | 8,58633045 | 0,69641944 | 0,48721664 | 0,50056504 | -7,2279923 |
| DNER | -0,0455558 | 8,72143839 | -0,6114609 | 0,54179661 | 0,54911819 | -7,2835778 |
| TRAIL | 0,00270721 | 7,5019649 | 0,02037769 | 0,98376847 | 0,98376847 | -7,4707003 |
| Abbreviations: logFC = logarithm of the fold change; AveExpr = average log-expression values; t = moderated t-statistic; P.Value = associated p-value; adj.P.Value = the p-value adjusted for multiple testing using Benjamini and Hochberg method; B = log-odds that the gene protein differentially expressed. | | | | | | |
